# How generalizable is the inverse relationship between social class and emotion perception?

**DOI:** 10.1371/journal.pone.0205949

**Published:** 2018-10-19

**Authors:** Christen M. Deveney, Stephen H. Chen, Jeremy B. Wilmer, Valerie Zhao, Hannah B. Schmidt, Laura Germine

**Affiliations:** 1 Department of Psychology, Wellesley College, Wellesley, MA, United States of America; 2 Institute for Technology in Psychiatry, McLean Hospital, Belmont, MA, United States of America; 3 Department of Psychiatry, Harvard Medical School, Boston, MA, United States of America; University of Texas Medical Branch at Galveston, UNITED STATES

## Abstract

Compared to individuals in lower positions of power, higher-power individuals are theorized to be less motivated to attend to social cues. In support of this theory, previous research has consistently documented negative correlations between social class and emotion perception. Prior studies, however, were limited by the size and diversity of the participant samples as well as the systematicity with which social class and emotion perception were operationalized. Here, we examine the generalizability of prior research across 10,000+ total participants. In an initial modest sample, (n = 179), Study 1 partially replicated past results: emotion identification correlated negativity with subjective social class (β = -0.15, 95% CI = [-0.28,-0.02]) and one of two objective social class measures (participant education β = -0.15, 95% CI = [-0.03,-0.01]). Studies 2–4 followed up on Study 1’s mixed results for objective social class in three much larger samples. These results diverged from past literature. In Study 2, complex emotion identification correlated non-significantly with participant education (β = 0.02, *p* = 0.25; 95% CI = [-0.01, 0.05], *n =* 2,726), positively with childhood family income (β = 0.03, 95% CI = [0.01,0.06], *n* = 4,312), and positively with parental education (β = 0.06, 95% CI = [0.04,0.09], *n =* 4,225). In Study 3, basic emotion identification correlated positively with participant education (β = 0.05, 95% CI = [0.02, 0.09]), *n* = 2,564). In Study 4, basic emotion discrimination correlated positively with participant education (β = 0.09, 95% CI = [0.05,0.13], *n* = 2,079), positively with parental education (β = 0.06, 95% CI = [0.02,0.09], *n* = 3,225), and non-significantly with childhood family income (β = 0.2, 95% CI = [0.01,0.07], *n* = 3,272). Results remained similar when restricting analyses to U.S.-based participants. Taken together, these findings suggest that previously reported negative correlations between emotion perception and social class may generalize poorly past select samples and/or subjective measures of social class. Data from the three large web-based samples used in Studies 2–4 are available at osf.io/jf7r3 as normative datasets and to support future investigations of these and other research questions.

## Introduction

A growing body of research indicates that social class influences numerous aspects of social behavior including the ability to identify the emotions of others (i.e., emotion perception). Specifically, across a number of studies, lower social class has been associated with better emotion perception [1–3]. According to emerging theories, the experiences of lower-class individuals (e.g., less access to material resources) necessitate greater interdependence which fosters increased attentiveness to others’ emotions [1, 4]. In contrast, individuals from higher social classes, whose material wealth and social status confer greater independence, may be less motivated to attend to others’ emotions [1, 4].

Despite the general consistency of these results, a major limitation of the research to-date has been the inconsistent operationalization of social class and emotion perception across studies. For example, objective social class has been operationalized using education attainment [1], income [5, 6], or composite indices of education and annual income [5, 6]. Subjective social class has been measured using the MacArthur Scale of Subjective Socioeconomic Status [7], experimental manipulation of subjective social class [1, 5, 6], and also by comparing the objective socioeconomic status (SES) rankings of participants in a dyadic interaction [5].

Measures of emotion perception also vary between studies. Some studies assess performance on varying standardized face emotion identification tasks [1]. Other studies examine how well participants judge a study partner’s emotions [1, 5]. In addition, emotion perception ability is sometimes measured using accuracy [1], and other times by the variability in emotion ratings [8]. The inconsistent operationalization of social class and emotion perception across studies, as well as the use of primarily modest-sized university samples, limits the generalizability of this research. As such, there is a distinct need to identify the specific processes of emotion perception that may be influenced by specific elements of social class, and there is a need to examine these processes in large and diverse samples.

### Present study

To examine the generalizability of previous research on social class and emotion perception, the present study examined different operationalizations of social class (e.g., subjective and objective; income and education) and emotion perception (e.g., identification of complex and basic emotions and emotion discrimination) in four independent studies. Following a previously-used protocol, Study 1 used an experimental design to examine relations between complex emotion identification ability and objective and subjective measures of social class. Study 2 expanded the correlational analyses of Study 1 to a larger sample and examined relations between complex emotion identification ability and objective measures of social class. In Studies 3 and 4, we examined whether the findings from Study 1 and 2 were due to the specific tasks used. Study 3 examined links between objective social class and identification of basic emotions. Study 4 examined associations between objective measures of social class and discrimination between emotional expressions. By capitalizing on the diverse international samples in Studies 2–4, we were able to explore whether relations between social class and emotion perception are unique to U.S. samples or whether they generalize across cultural boundaries.

## Study 1

Following a protocol used in previous research [1], Study 1 examined relations between face emotion identification and two measures of social class: objective social class (measured by education and income) and experimentally-manipulated subjective social class. Similar to previous research, we tested two hypotheses: (1) higher subjective social class would cause poorer emotion perception; and (2) higher objective social class–operationalized as income and education–would be associated with lower emotion perception. The research plan and analyses were pre-registered with the Open Science Framework at the Center for Open Science (experimental manipulation: https://osf.io/vab4f/ and correlational analyses: https://osf.io/dgnhs/).

## Study 1 method

### Participants

One hundred ninety-two adults (ages 19–71) were recruited online using Amazon Mechanical Turk (MTurk) to participate in a study that “examines how well participants recognize feelings and individuals from pictures.” Individuals were eligible for participation if they (1) had approved HITs (Human Intelligence Tasks) greater than 100; (2) had a HIT approval rating of 95% or higher; (3) were located in the United States; and (4) were 18 years of age or older. Individuals who opted to participate were redirected to an online consent form using Qualtrics. Due to MTurk policies, only participants who completed the entire study received $3 for participation. The project was approved by the Institutional Review Board at Wellesley College.

### Questionnaires

Participants self-reported demographic information and completed four standard questionnaires: (1) the Agreeableness Scale of the Big Five Personality Inventory [9]; (2) the Positive and Negative Affect Scale [10]; (3) the Self-Monitoring Questionnaire [11], and (4) the Autism Questionnaire [12].

### Subjective measure of social class

Participants completed the subjective social class manipulation used by Kraus et al. (2010; Study 3). Participants viewed an image of a ladder whose rungs represented “where people stand in the United States.” Participants were randomly assigned to compare themselves to individuals on either the highest (i.e., low social class manipulation) or lowest (i.e., high social class manipulation) rung of the ladder and were asked to write about a hypothetical interaction with this individual. Next, participants ranked their perceived SES on the same ladder. This resulted in two separate measures of subjective social class: (1) status manipulation condition, and (2) participant ladder ranking.

### Objective measure of social class

Objective social class was measured using two variables: highest level of education and annual income. Participants who completed a 4 year college degree were categorized in the high objective social class category and individuals who had not completed a 4 year degree were categorized in the low objective social class category. Participants reported their annual income using the following categories: (1) under $10,000; (2) $20,000-$29,999; (3) $30,000-$39,999; (4) $40,000-$49,999; (5) $50,000-$74,999; (6) $75,000-$99,999; (7) $100,000–150,000; (8) more than $150,000.

### Emotion perception task

Emotion perception was assessed using the Reading the Mind in the Eyes Test (RMET) [13]. All participants viewed 36 images of eyes expressing different emotions. Each image was accompanied by four emotion words. The definitions for each emotion word were displayed by hovering a cursor over the word. Participants were instructed to choose the emotion word that best matched the emotion in the image.

### Additional tasks

To account for the language demands of the RMET, participants also completed a vocabulary test [14]. The Famous Faces Task [15] was used as a filler task (see procedure below) during which participants were asked to identify 10 famous individuals (e.g., George Clooney).

### Procedure

Participants followed the same procedure as Kraus et al. (2010; Study 3): subjective social class manipulation, RMET, filler task (Famous Faces Task), and the agreeableness scale. Additional measures used in this study were then completed in the following order: PANAS, Vocabulary Test, Autism Questionnaire, Self-Monitoring Scale, demographic questions, and the open-ended interaction questions. Three “catch” items were interspersed throughout the additional measures in order to assess participant attention and accuracy.

### Statistical analyses

Participants were excluded from analyses if they: (1) failed two of three “catch” items; (2) indicated “little to no English proficiency” or did not complete this item; (3) missed one item or more item on either the RMET or Agreeableness Scale; (4) did not complete the subjective socioeconomic status rating; or (5) responded “other” or declined to answer the gender identity question.

The association between subjective social class and emotion identification ability was tested using an analysis of covariance (ANCOVA) with *Condition* (low/high subjective social class) as the between-participants factor, gender and agreeableness as covariates (as in past research), and RMET scores as the dependent variable. A hierarchical regression was used to test associations between ladder ratings and RMET scores using the same covariates. Finally, we tested whether the manipulation successfully altered subjective social status using an ANOVA to compare ladder ratings between the two conditions (*note*: this analysis was not included in the pre-registration).

Relations between objective social class and emotion identification ability were evaluated using a series of multiple hierarchical regressions with two separate indicators of objective social class as predictor variables (income and completion of 4 year college degree). Covariates included age, gender, and vocabulary to account for the language demands of the RMET. This analysis was repeated using age and age^2^ as additional covariates, because prior data suggest that RMET performance improves and then declines over the lifespan [16]. Analyses with income and highest level of education were restricted to participants age 25 and older (N = 148).

## Study 1 results

### Participants

Data from 13 (6.8%) participants were excluded (3 missed more than one catch item, 1 did not complete the RMET and several other questionnaires; 9 responded “other” or “prefer not to answer” on the gender identity question), leaving 179 individuals for analysis. Although the pre-registration did not include an exclusion for gender non-binary responses, we were unable to run the appropriate statistical analysis if this small number (N = 9) of participants were included. Eighty-three individuals were randomized to the low subjective social class condition and 96 individuals to the high subjective social class condition. The groups did not differ on any demographic variables, nor on accuracy on the Famous Faces and vocabulary tasks (See Tables 1 and 2).

**Table 1:** Study 1 participant age and task performance in each social class manipulation condition.

	Low Social Class Condition (N = 83)		High Social Class Condition (N = 96)			
Characteristic	*M*	*SD*	*M*	*SD*	*Statistic*	*p*
Age (years)	34.46	9.7	35.33	11.9	*F*(1,177) = 0.28	*p* = .60
Famous Faces Score	5.55	2.5	5.22	2.3	*F*(1,177) = 0.89	*p =* .35
Vocabulary Score	14.57	3.7	14.81	3.7	*F*(1,177) = 0.20	*p* = .66
Agreeableness	47.92	8.2	46.39	8.8	*F*(1,177) = 1.43	*p* = .23

**Table 2:** Study 1 participant age, ethnicity, and education status in each social class manipulation condition.

	Low Social Class Condition (N = 83)		High Social Class Condition (N = 96)			
Characteristic	N	%	N	%	*Statistic*	*p*
Female	52	62.7%	53	55.2%	χ^2^(1) = 1.02	*p* = .31
Non-Minority Ethnicity	59	71.1%	71	74.7%	χ^2^(1) = 0.30	*p* = .58
Four-year college degree	42	50.6%	36	37.5%	χ^2^(1) = 3.11	*p* = .08

### Subjective social class and emotion perception

**Fig 1:**
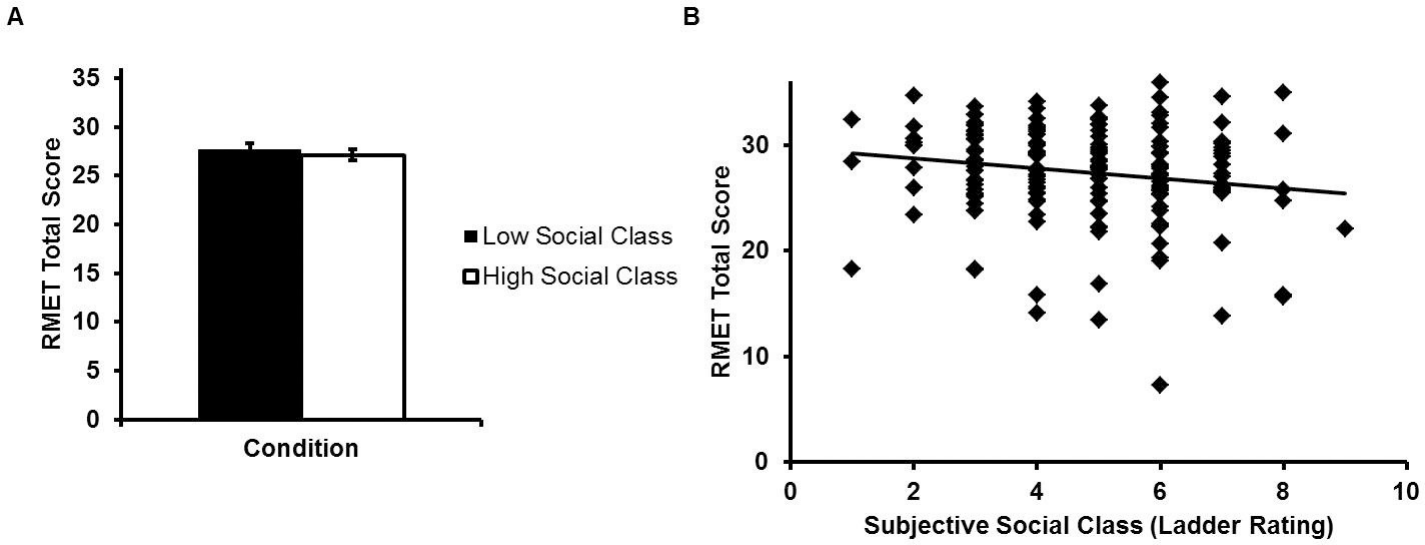
Panel A: Face emotion identification ability as a function of subjective social class manipulation condition in Study 1. RMET scores have been corrected for gender and agreeableness. Error bars reflect standard errors. Panel B: Participant RMET performance as a function of their subjective social class rankings (higher ladder ratings = higher social class). RMET scores have been adjusted for vocabulary, agreeableness, and gender.

RMET scores did not differ between individuals in the high and low subjective social classes (*F*(1,175) = 0.53, *p* = 0.47,η_p_^2^ = 0.003; Fig 1A). However, this may be due to a failure of the subjective social class manipulation, as ladder ratings did not differ between the two conditions (*F*(1,177) = 0.51, *p* = 0.48, η_p_^2^ = 0.003; M_low_ = 4.83, SD_low_ = 1.71;M_high_ = 4.66, SD_high_ = 1.56).

Lower social class ladder rating was related to higher RMET scores (Fig 1B; Table 3). The relationship remained significant when age variables were included as covariates (β = -0.15, *p* = 0.02; S1 Table).

**Table 3:** The relationship between subjective social class (ladder ratings) and rmet performance in study 1 after accounting for gender, vocabulary, and agreeableness.

Predictor		Subjective Social Class (Ladder Ratings)	Education	Income
Gender	*B*	0.28	0.53	0.98
	95% CI	[-1.12, 1.68]	[-0.95, 2.00]	[-0.50, 2.45]
Vocabulary	*B*	0.66***	0.68***	0.69***
	95% CI	[0.48, 0.85]	[0.47, 0.88]	[0.49, 0.90]
Agreeableness	*B*	0.09*	0.08	0.10*
	95% CI	[0.01, 0.17]	[0.00, 0.17]	[0.01, 0.18]
Social Class	*B*	-0.49	-1.37	-0.21
	95% CI	[-0.91, -0.07]	[-2.80, 0.6]	[-0.58, 0.17]
	*N*	179	148	144
	R^2^	.26	.24	.25
	*F*	15.02***	11.54***	11.67***

*Note*. CI = confidence interval.

*p ≤ .05.

** *p* ≤ .01.

*** *p* < .001.

### Objective social class and emotion perception

**Fig 2:**
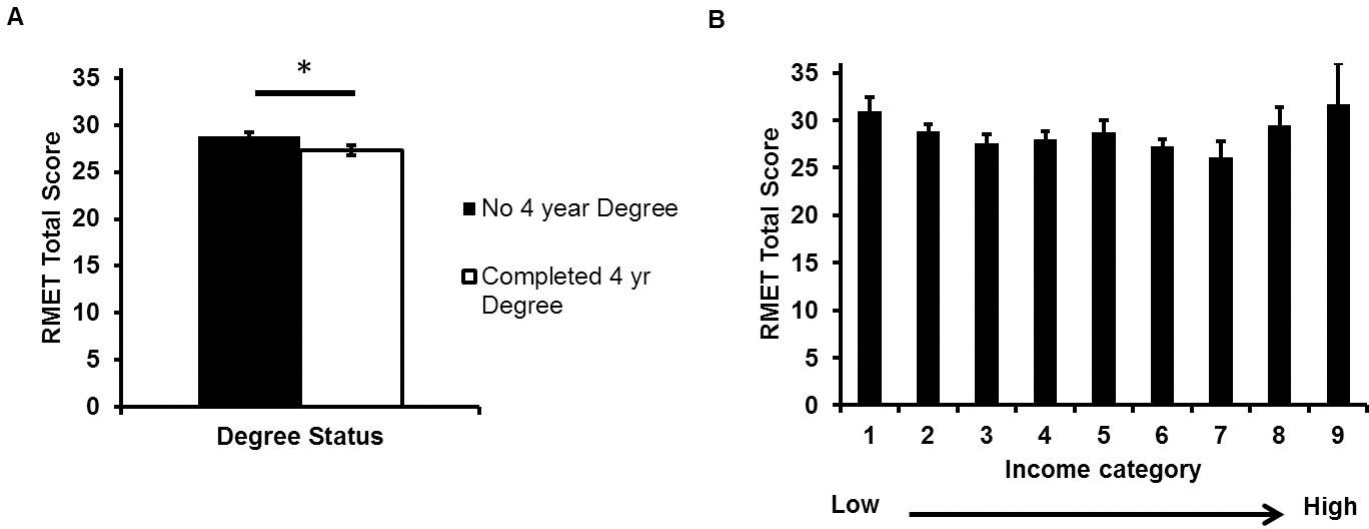
Face emotion identification ability as a function of objective social class after accounting for covariates in Study 1. Panel A: Participants who did not complete a four-year college degree performed better on the RMET than participants who completed a four-year degree. However, this was only significant when age variables were included as covariates. Panel B: Participant self-reported income was unrelated to RMET performance regardless of covariates. *Note*: RMET scores corrected for vocabulary, agreeableness, gender, age, and age^2^. Income categories were as follows: 1 = Under $10,000 (N = 9); 2 = $10,000–19,999 (N = 27); 3 = $20,000–29,999 (N = 24); 4 = $30,000–39,999 (N = 28); 5 = $40,000–49,999 (N = 10); 6 = $50,000–74,999 (N = 34); 7 = $75,000–99,999 (N = 6); 8 = $100,000–150,000 (N = 5); 9 = Above $150,000 (N = 1). Error bars reflect standard errors.

Neither measure of objective social class was significantly associated with RMET score (Table 3). When the age variables were included as covariates, completion of a 4-year college degree (β = -0.15, *p* = 0.04), but not income (β = -0.11, *p* = 0.12), was significantly associated with poorer RMET performance (Fig 2; S1 Table).

### Factors contributing to the relationship between social class and emotion perception

We conducted exploratory analyses to examine why the subjective social class manipulation was unsuccessful and why objective social class was not consistently related to RMET scores in all models. First, prior evidence suggests that autism symptoms are higher in MTurk samples than in community and undergraduate samples [17]. Given links between autism spectrum disorders and impaired social awareness, including emotion recognition deficits [18], we wanted to rule out the possibility that responses to the social class manipulation and/or performance on the RMET were impacted by the presence of participants with elevated autism spectrum symptoms. Therefore, we repeated each of the primary analyses after excluding individuals whose AQ scores exceeded the clinical threshold [19] (low_excluded_ N = 25; high_excluded_ N = 21). Consistent with the original findings, the subjective social class manipulation conditions did not differ on RMET performance or ladder rating scores. Subjective ladder ratings were no longer related to RMET scores. In addition, neither income nor education was associated with RMET performance. College education was significant at the trend level (*p* = 0.06) only when age and age^2^ were included as covariates.

Second, in order to adhere to prior analytic strategies [1]), vocabulary was not included as a covariate in the subjective social class ANCOVA reported above. However, because vocabulary was a robust predictor of RMET performance in the objective social class analyses, we repeated the ANCOVA after including vocabulary as a covariate. No group differences emerged (*F*(1,174) = 1.19, *p* = 0.28, η_p_^2^ = 0.01).

## Study 1 conclusions

In contrast to previous work [1], a downward social class manipulation did not positively impact RMET performance in the present sample. This could be because the social class manipulation did not impact subjective social class in our study, thus raising questions about whether this manipulation is effective under different testing conditions (online versus in person) and varying participant ages (varied adult ages versus only undergraduates). Despite the manipulation failure, higher ladder rankings were related to poorer emotion identification, providing some support for prior work.

Our results produced some support for the hypothesized relations between objective measures of social class and emotion perception. Consistent with prior research, lower education—as operationalized by lack of a four-year college degree—was associated with better emotion identification; however, this relationship emerged only when vocabulary and age were controlled for, which had not been done in prior studies. Contrary to prior findings, income was not related to emotion identification ability.

Given the weak support for relations between education level and emotion perception, our next step was to examine these associations in a larger online sample. As the online social class manipulation failed to impact subjective social class, this experimental aspect of the protocol was not included in subsequent studies. Therefore, the remaining studies focused on relations between objective social class and emotion perception.

## Study 2

Using a large independent sample (N = 5,187), Study 2 tested whether three different measures of lower objective social class were associated with higher emotion perception, as measured by the RMET. In contrast to participants from Study 1, Study 2 also included a large group of individuals from outside of the U.S. Initial analyses of the full sample allowed us to examine the broad generalizability of prior results by testing whether the negative correlations between social class and emotion perception generalized to non-U.S. cultures. A subsequent set of analyses then tested the replicability of prior results in a subsample of participants whose sociodemographic characteristics mirrored those of participants in the prior work. The analyses and research plan were similar to the correlational analyses in Study 1. As these data were collected previously and for a different purpose [16], they included some but not all of the same measures as Study 1. The data from Study 2 are available at osf.io/jf7r3.

## Study 2 methods

### Participants

Data were collected via TestMyBrain.org (TMB), a cognitive testing website that seeks to induce attentive and motivated participation by providing immediate performance feedback at the end of each battery. TMB tends to attract a diverse, international sample of participants [15]. TMB-collected data have been shown to mirror data collected in the lab on multiple quality measures: performance means, performance variances, and test reliabilities [20]. The present study involves data collected between June 2013 and January 2014, via a test battery called “The Social Mind and Life Experiences.” Our analyses were conducted on the 5,187 individuals (of 6,723 total) who were between the ages of 18–90, who provided gender information, and who completed both the vocabulary measure used in Study 1and the RMET. The mean age of this sample was 30.6 years (SD = 12.5), 56% of the sample was female, 46% were non-native English speakers, 61% reported a non-U.S. country of origin (see S2 Table for a list of the specific countries represented), 32% reported non-European/White ethnicity, 47% did not have a college degree, and 12% reported no college education.

The social class analyses described below included only those participants reporting education and income data. Analyses involving participants’ education were also limited to individuals who reported being at least 25 years of age in order to accommodate completion time for a four-year college degree. This data collection was approved by the Harvard University Institutional Review Board. Participants signed informed consent and data were collected anonymously.

### Measures

#### Objective tests

Vocabulary and face emotion identification were measured using the same tests as Study 1 (Vocabulary: [14]; RMET: [13]), although definitions for the response choices were not available on this version of the RMET. Participants also completed a change detection test (not analyzed here).

#### Questionnaires

Participants reported demographics and completed a childhood adversity questionnaire that included a question on child family income and parental education (for full details of questionnaire, see [16]). Participants reported their highest level of education as: (1) middle school; (2) high school/secondary school; (3) some college/university; (4) bachelor’s degree; (5) graduate degree; (6) none of the above or I’d rather not say. As previous investigations of social class and emotion perception have used attainment of college degrees as a marker of higher social class (Kraus et al., 2010), and to follow the operationalization of social class used in Study 1, participants choosing options 4 and 5 (completion of a four-year college degree) were categorized as high social class. Those choosing options 1–3 (no completion of a four-year college degree) were categorized as low social class. Participants who selected (6) were excluded from the education analyses.

The income question, designed for use in international samples, was: “Compared to the other families in the same country or part of the country, what was your family's income? (1) Much lower than the average household [scored -2]; (2) Somewhat lower than the average household [scored -1]; (3) Similar to the average household [scored 0]; (4) Somewhat higher than the average household [scored 1]; (5) Much higher than the average household [scored 2]; (6) I don't know; (7) I'd rather not say.” These data were treated as an ordinal variable with higher scores reflecting higher social class. Participants who answered selected (6) or (7) were excluded from the income analyses.

The parental education question was “What is the highest degree your mother (father) earned? Responses to this question were coded as: (0) less than high school; (1) high school diploma or certificate; (2) bachelor’s degree; (3) master’s degree; (4) doctorate; (5) professional; (-1) none of the above; I’d rather not say. To maintain consistency with the operationalization of social class used in the prior studies, both maternal and paternal education variables were dichotomized into (1) completion of a four-year college degree (response options 2, 3, 4, or 5) or (2) non-completion of a four-year-college degree (options 0, or 1). Participants who answered “I don’t know” or “I’d rather not say” were excluded from further analyses. The dichotomized variables for maternal and paternal education were combined into a single parental education variable: (0) neither parent has a four-year college degree; (1) at least one parent has a four-year college degree; (2) both parents have four-year college degrees. When education was reported for at least one parent but not another, parental education was calculated based on the highest degree attained by the parent with education data. Parent education was treated as an ordinal variable. Lowest social class was operationalized as neither parent having a four-year college degree. Highest social class was operationalized as both parents having completed a four-year college degree.

### Procedure

Participants completed the questionnaires and tasks in the following order: (1) age, education, and gender questions; (2) change detection task; (3) vocabulary task; (4) RMET; (5) childhood adversity questionnaire; and (6) ethnicity, country of origin, and native language questions.

### Statistical analyses

We conducted three hierarchical linear regression analyses to examine whether each social class variable was associated with lower face emotion identification performance. These analyses controlled, respectively, for gender and vocabulary (Analysis 1); gender, vocabulary, age, and age^2^ (Analysis 2), and mirrored the correlational analyses conducted for Study 1. Unlike the analyses in Study 1, Study 2 analyses did not control for agreeableness (which was not available in this data set). Due to differences in data available for each social class variable and because the education analyses were restricted to individuals age 25 years and older, the number of participants in each analysis varied (N_education_ *=* 2,726; N_income_ *=* 4,312; N_parental education_ *=* 4,225).

The expanded participant pool of Study 2 allowed us to examine whether results of Study 1 and those of prior studies could be generalized to a sample that included non-U.S. participants, non-White/European individuals, and non-native English speakers. First, participants in prior studies–including the present Study 1 –were recruited from within the United States. In contrast, the majority of Study 2 participants reported growing up outside of the United States. Second, prior work has documented face-processing differences for within- and between-group stimuli [21]. The RMET stimuli consist of European/White individuals, raising the possibility that the “other race effect” may have obscured the relationship between social class and emotion perception. Third, because the RMET stimuli involve complex emotions, it is possible that language barriers may have impacted RMET performance. To address these obstacles, we conducted additional hierarchical linear regression analyses using a specific subset of participants who reported the United States as their country of origin, indicated ethnicity as European/White, and reported being a native English speaker (N_education_ = 990; N_income_ = 1,278; and N_parent education_ = 1,267). This regression included gender, vocabulary, age, and age^2^ as covariates.

## Study 2 results

**Fig 3:**
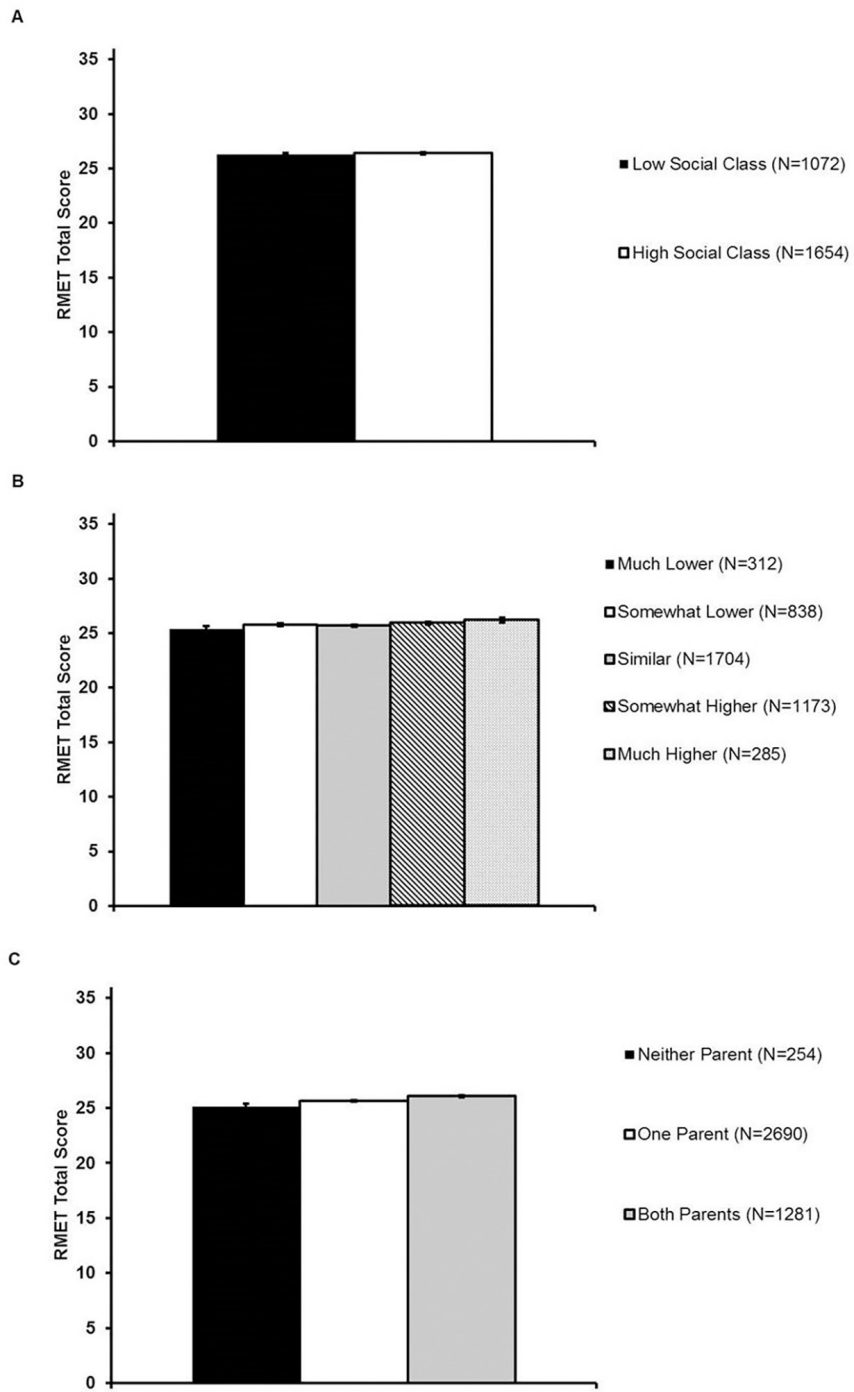
Face emotion identification ability in the full sample as a function of objective social class in Study 2. Panel A: RMET performance was similar between individuals who completed a four-year degree and those who did not complete a four-year degree. Panel B: Higher participant childhood family income was associated with higher RMET scores. Panel C: Higher parental education was associated with higher RMET scores. Note: All RMET scores corrected for vocabulary, agreeableness, gender, age, and age^2^. Error bars reflect standard errors.

Female gender and higher vocabulary scores were associated with better RMET performance in every regression model (Table 4). The predictions from prior literature—that lower education and lower income would be associated with higher RMET performance—did not hold in any analysis (Fig 3; Table 4; S3 Table). Contrary to prediction, higher education and higher income were associated with higher RMET performance, and the income and parental education effect sizes reached statistical significance. The relationship between education and RMET performance was significant (β_education_ = 0.07; *p* = 0.03; β_parent education_ = 0.08; *p* = 0.002) after the sample was restricted to individuals reporting English as their native language and European/White ethnicity (see S4 Table). However, the income association was no longer significant (β_income_ = 0.03; *p* = .19; S4 Table).

**Table 4:** The relationship between different measures of social class and rmet performance in Study 2.

Predictor		Participant Education	Family Income	Parental Education
Gender	*B*	1.36***	1.41***	1.37***
	95% CI	[1.06, 1.67]	[1.16, 1.65]	[15.56, 16.72]
Vocabulary	*B*	0.55***	0.54***	0.55***
	95% CI	[0.52, 0.59]	[0.51, 0.57]	[0.52, 0.58]
Social Class	*B*	-0.18	0.15*	0.53***
	95% CI	[-0.13, 0.48]	[0.03, 0.27]	[0.31, 0.76]
	*N*	2,726	4,312	4,225
	R^2^	.28	.26	.27
	*F*	353.30***	506.78***	520.32***

*Note*. CI = confidence interval.

*p ≤ .05.

** *p* ≤ .01.

*** *p* < .001

## Study 2 conclusions

Contrary to previous research and findings from Study 1, the associations between lower objective social class, measured via education and income, and better emotion perception measured via the RMET, were not observed within a larger, independent, and more diverse sample. Instead, the opposite pattern of findings emerged, with lower social class being associated with poorer RMET performance. The discrepancy between these findings and previously published work cannot be attributed solely to cultural differences between the full sample and previously published work. Analyses of the subsample consisting of U.S.-originated native English speakers of White/European ethnicity demonstrated a similar link between lower social class and poorer emotion perception. Taken together with Study 1’s finding that lower education was associated with higher face emotion identification only after including certain covariates, these results suggest that the tendency of those with lower objective social class to perform better on tests of emotion perception may be less robust than previously stated. However, the relationship between social class and emotion perception performance varied depending on the specific variable used to operationalize social class and the sociodemographic characteristics of the sample.

This dataset did not include an agreeableness measure. Therefore, we cannot rule out the possibility that differences in agreeableness, which correlated positively with face emotion identification in Study 1 and prior work, were lower in those with lower education, income, or parental education. That possibility, however, seems unlikely since those with lower education rated themselves as slightly more agreeable, not less, in Study 1.

## Study 3

The results of Studies 1 and 2 did not provide strong support for associations between higher social class and lower emotion perception. In Study 3, we sought to extend the literature by examining whether these findings were influenced by the specific measure of emotion perception used in those studies. Although the RMET is a well-established measure of face emotion identification, it is unique in its use of complex emotions and is closely tied to vocabulary. Therefore, Study 3 explored whether lower education was associated with better emotion perception on a more traditional face emotion identification task that used basic emotions. As in Study 2, we sought to test these relationships using a large and diverse sample. Also similar to Study 2, analyses were conducted with a subsample of participants chosen to be more sociodemographically similar to prior studies. The analyses and research plan were similar to the correlational analyses in Study 2. As these data were collected previously and for a different purpose [22], they included some but not all of the same measures as Studies 1 and 2. The data from Study 3 are available at osf.io/jf7r3.

## Study 3 methods

### Participants

Data for Study 3 were collected via TMB as part of a test battery called ‘Recognizing Emotions’ between June 2017 and October 2017. Analyses for Study 3 were limited to data from the 2,564 individuals (of 6,265 total) ages 25–90 years who completed the emotion identification task described below and provided gender, education, and native language information. Mean age was 39.31 years (SD = 12.29), 62.1% of the sample was female, 25.1% were non-native English speakers, 20.6% reported non-European/White ethnicity (see S2 Table for a list of countries represented in the participant sample), and 35.0% did not have a four-year college degree. Data collection was approved by the Harvard University Institutional Review Board Participants signed informed consent and data were collected anonymously.

### Measures

#### Emotion identification task

The multiracial emotion matching test is an emotion identification test that asks participants to indicate whether each image is happy, sad, angry, or fearful. Participants view 48 images individually and have up to 10 seconds to make a choice before the next trial is presented. Face stimuli include several races/ethnicities (44% European/White; 29% Asian; 25% African American/Black; and 2% Hispanic/Latino), a range of adult ages, and equal proportions of men and women. Stimuli in this test were selected, based on pilot testing, to reduce the ceiling effects that limit sensitivity and validity of many existing emotion identification tests [23]. To develop this test, we recruited actors from across a range of ages and race/ethnicities, from the [masked for blind review] theater, as part of the Act Out for Brain Health project. Images were taken from video clips of actors portraying different emotions. An initial set of 146 images were selected to create an item bank. Images were drawn from this item bank and data was collected from a pilot sample (N = 8,309) of participants who each saw a subset of 37–53 images. The final test used in the present study included 48 images that were selected, based on the pilot data set, to meet the following criteria: (1) images with consistent judgments of a single emotion (minimum accuracy 60%; chance is 25%); (2) varying levels of difficulty for each emotion (between 60 and 100% accuracy); (3) robust contribution to internal reliability (i.e., high correlations of scores on individual items with total score on the remaining test items); and (4) preservation of the diversity of actors and faces. Because our interest in the present study was overall emotion perception, we used total score (maximum 48) as the dependent variable.

#### Questionnaires

Participants reported age, gender, education, ethnicity, and native language. The education question was identical to the one used in Study 2 Participants choosing options 4 (bachelor’s degree) or 5 (graduate degree) were categorized as high social class and those choosing options 1–3 (middle school, high school/secondary school, or some college/university) were categorized as low social class.

### Procedure

Participants completed questionnaires and tasks in the following order: (1) age and gender questions; (2) face emotion identification task; (3) schizotypal personality disorder questionnaires (not analyzed here; see [22]); (4) social networks questionnaire (not analyzed here see [22]); and (5) education, ethnicity, and native language questions.

### Statistical analyses

Statistical analyses were similar to those used in Study 2, with small changes reflecting slight methodological differences between the studies. First, vocabulary was not included as a predictor because the emotion identification task in this study did not contain the same vocabulary demands as the RMET. Instead, we included native language (English/non-English) as a covariate. Second, the age covariates were included in the first hierarchical linear regression analysis in order to reduce the number of statistical analyses conducted. Therefore, the primary analysis tested whether higher education was associated with lower face emotion identification performance after controlling for gender, native language, age, and age^2^.

Similar to Study 2, the participants in this sample were more diverse than those in Study 1 and many prior studies of emotion perception in the literature. Therefore, we repeated the hierarchical linear regression analysis after limiting the sample to participants who reported their ethnicity as European/White and who reported being a native English speaker (N = 1,486). This regression included gender, age, age^2^ as covariates.

## Study 3 results

### Objective social class and emotion perception

**Fig 4:**
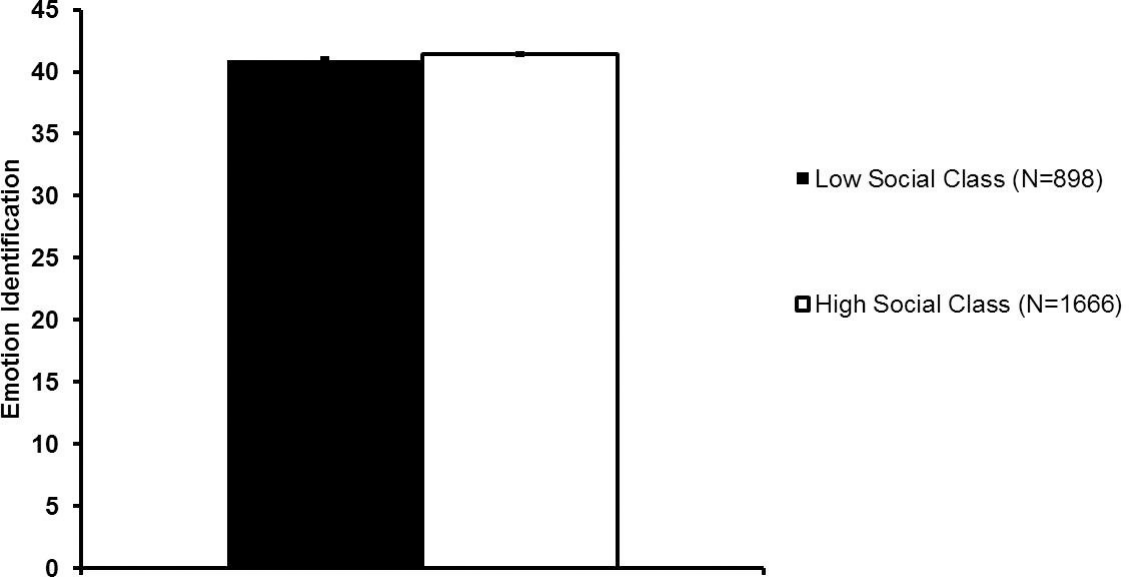
Face emotion identification ability as a function of education status in Study 3. Emotion identification scores for the entire sample following correction for gender, native language, age, and age^2^. Error bars reflect standard errors. Maximum score = 60.

Contrary to predictions, lower education was related to poorer face emotion identification (Fig 4; Table 5), even after accounting for age, native language, and gender, which were all significant predictors of emotion perception. This relationship was only significant at the trend level (β = 0.05; *p* = 0.05) after the sample was restricted to individuals reporting English as their native language and European/White ethnicity (N = 1,486; see S5 Table).

**Table 5:** The relationship between participant education and emotion identification ability in Study 3.

Predictor		Participant Education
Gender	*B*	0.90***
	95% CI	[0.58, 1.21]
Age	*B*	0.12**
	95% CI	[0.04, 0.20]
Age^2^	*B*	-0.002***
	95% CI	[-0.01, 0.00]
Native Language	*B*	0.81***
	95% CI	[0.45, 1.16]
Social Class	*B*	0.45**
	95% CI	[-0.13, 0.76]
	*N*	2,564
	R^2^	0.03
	*F*	17.95***

*Note*. CI = confidence interval.

*p ≤ .05.

** *p* ≤ .01.

*** *p* < .001

## Study 3 conclusions

Higher social class, operationalized as the completion of a four-year college degree, was related to better performance on a traditional face emotion identification task that used basic emotions and did not have the same language demands as the RMET. Considered with the findings from Studies 1 and 2, the present findings suggest that the relationship between social class and emotion perception varies depending on the participant population and the measure of emotion perception. In the large and diverse sample used in Study 3, lower social class is associated with poorer identification of basic emotions from facial stimuli. Associations between lower social class and poorer emotion identification only existed at a trend level in the restricted sample. We note that this dataset did not include a measure of income or parental education. Therefore, we cannot determine whether these other measures of social class are related to performance on this traditional face emotion identification task.

## Study 4

Prior research on social class and emotion perception, including the three studies of the current investigation, has focused on the ability to identify the emotional expression depicted in a single image. However, emotion perception also includes discriminatory abilities of determining whether two individuals are expressing the same, or different, emotions. The final study explored whether lower social class would be related to better emotion discrimination ability. The analysis and research plan were similar to the correlational analyses completed in Studies 2 and 3, and also used data collected previously for a different purpose [16].

## Study 4 methods

### Participants

Data for Study 4 were collected via the same TMB test battery (“The Social Mind and Life Experiences”) as Study 2 (see also [16]). However, as described in [16], participants who clicked on the link were given different emotion perception tasks depending on when they completed the study. The RMET task reported in Study 2 and the face emotion discrimination task reported in Study 4 were completed at different times (for additional details see [16]). Therefore, the participant sample does not overlap between studies. The data in Study 4 were collected between August and December 2013. Our analyses for Study 4 were conducted on the 3,859 (of 6,723 total) individuals who were between18-90 years of age, who provided gender and native language information, and who completed both an emotion discrimination and an identity discrimination task (described below). The mean age of this sample was 30.2 years (SD = 12.1), 59% of the sample was female, 50% were non-native English speakers, 65% reported non-U.S. country of origin (see S2 Table for a list of countries represented in the participant sample), 31% reported non-European/White ethnicity, 48% did not have a college degree, and 12% reported no college experience.

Analyses included data from participants who provided education and income data. Consistent with Studies 2 and 3, we further limited to the education analysis to individuals who were at least 25 years of age. This data collection was approved by the Harvard University Institutional Review Board. Participants signed informed consent and data were collected anonymously.

### Measures

#### Emotion and face identity discrimination task

The Queen Square Face Discrimination Test: Emotion and Identity Subtests [24] were used as measures of face emotion and face identity discrimination ability, respectively. During both tasks, participants viewed two female faces presented in quick succession (500ms each). The images consisted of sad, fearful, disgusted, angry, happy, and surprised images from the Ekman and Friesen (1976) image set [25]. During the *emotion identification* task, participants pressed a button to indicate whether the faces expressed the same or different emotion. During the *face identity* task, participants pressed a button to indicate whether the faces depicted the same or different individuals. Consistent with Study 3, we sought to understand overall emotion perception ability. Therefore, the primary measure of emotion perception was the total number of correct trials on the emotion identification task (maximum = 72). Total score on the face identity task was used to assess participants’ ability to distinguish between different individuals and to control for the working memory component associated with both tasks.

#### Questionnaires

Education and income questions and the operationalization of social class were the same as those used in Study 2.

### Procedure

After providing informed consent, participants completed demographic questions, followed by the emotion and face identity discrimination tasks.

### Statistical analyses

Statistical analyses paralleled those of Study 3, differing only in the inclusion of face identification task performance as a covariate. This allowed us to evaluate whether social class was related to emotion discrimination ability after accounting for ability to distinguish between different individuals and for working memory. The primary analysis consisted of three hierarchical linear regression analyses testing whether each social class variable was associated with face emotion expression discrimination performance after controlling for, respectively, gender, age, and age^2^, native language (non-English/English), and face identity discrimination performance (Analysis 1). Due to differences in data available for each social class variable, and because the education analyses were restricted to individuals aged 25 years and older, the size of each sample varied across analyses (N_education_ *=* 2,079; N_income_ *=* 3,272; N_parental education_ *=* 3,225).

As with Study 3, hierarchical linear regression analyses were repeated after restricting the dataset to the subset of participants who reported themselves to be native English speakers of European/White ethnicity (N_education_ = 953; N_income_ = 892; N_parent education_ = 1,317). Because the samples were limited to native English speakers, native language was removed from these regression models (Analysis 2). The data from Study 4 are available at osf.io/jf7r3.

## Study 4 results

**Fig 5:**
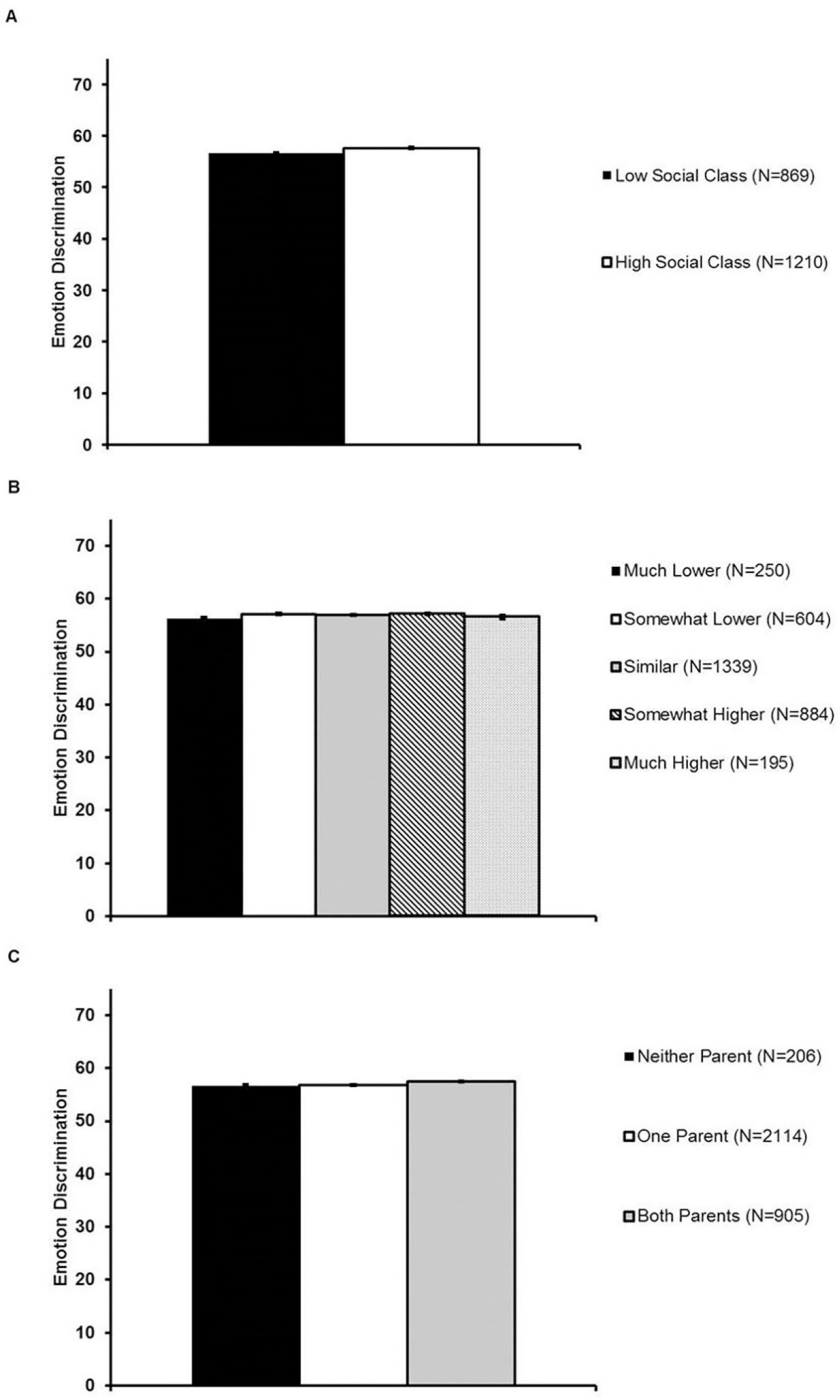
Face emotion discrimination ability in the full sample as a function of social class in Study 4. Panel A: Emotion discrimination scores for the entire sample were higher among individuals who completed a four-year degree compared to those who did not complete a four-year degree. Panel B: Higher participant childhood family income was not associated with emotion discrimination scores. Panel C: Higher parental education was associated with higher emotion discrimination scores. Note: All emotion discrimination scores corrected for native language, gender, age, and age^2^. Error bars reflect standard errors.

Consistent with Study 3, lower education, but not family income, was associated with poorer emotion discrimination, even after accounting for gender, age, native language, and performance on the face identity task (Fig 5; Table 6). The relationship between lower social class (operationalized using education variables) and poorer emotion perception remained significant when the sample was restricted to native English speakers of self-reported European/White ethnicity (see S6 Table).

**Table 6:** The relationship between different measures of social class and emotion discrimination ability in Study 4.

Predictor		Participant Education	Family Income	Parental Education
Gender	*B*	0.79***	0.60**	0.64***
	95% CI	[0.35, 1.24]	[0.24, 0.96]	[0.28, 1.00]
Age	*B*	0.04	0.04	0.06
	95% CI	[-.08, 0.16]	[-.03, 0.12]	[-.01, 0.14]
Age^2^	*B*	0.00	0.00	0.00
	95% CI	[-0.002, 0.001]	[-0.001, 0.001]	[-0.001, 0.001]
Native Language	*B*	0.29	0.24	0.28
	95% CI	[-0.18, 0.75]	[-0.12, 0.61]	[-.08, 0.64]
Identity Discrimination	*B*	0.36***	0.36***	0.36***
	95% CI	[0.32, 0.39]	[0.34, 0.39]	[0.33, 0.39]
Social Class	*B*	0.99***	0.09	0.56**
	95% CI	[0.56, 1.41]	[-0.09, 0.26]	[0.24, 0.87]
	*N*	2,079	3,272	3,225
	R^2^	.20	.19	.19
	*F*	84.83***	124.94***	127.02***

*Note*. CI = confidence interval.

*p ≤ .05.

** *p* ≤ .01.

*** *p* < .001.

## Study 4 conclusions

Consistent with Studies 2 and 3, lower social class was related to poorer performance on the face emotion perception task when social class was operationalized using education variables. Specifically, not completing a four-year college degree and having parents without four-year college degrees was related to poorer ability to discriminate between the emotional expressions of two individuals. This relationship was robust, with social class (participant education and parental education) predicting face emotion discrimination ability after accounting for covariates, including performance on a face identity discrimination task. The relationship remained significant even when the participant sample was restricted to individuals self-reporting White/European ethnicity and English as a native language. Together with Study 3, these findings suggest that, in contrast to previously published studies, higher, not lower, social class is linked to enhanced ability to identify and discriminate between emotional faces when social class is operationalized by the participant’s education and parental education background.

Unlike Studies 1–3, the emotion perception task in Study 4 required participants to remember the first emotional expression in order to decide whether the second image depicted the same emotional expression. Therefore, the task included a working memory component. Because this same working memory component was present in the face identity task, controlling for performance on this task suggests that the relationship between higher social class and better emotion discrimination is not due to working memory. However, future research using a validated neuropsychological measure of working memory would provide further insight on this matter.

## General discussion

The generalizability of the existing literature on social class and emotion perception [1–3] is limited by inconsistent variable operationalization and modestly-sized datasets. We sought to examine this generalizability through a pre-registered study (Study 1) supplemented by data from three large independent samples (Studies 2–4) and various operationalizations of social class and emotion perception. A summary of the primary study findings can be found in Table 7. Overall, the findings suggest that the relationship between social class and emotion perception may depend on how these variables are operationalized and also on the sociodemographic composition of the sample.

**Table 7:** Summary of findings studies 1–4.

	Task	Sample	Subjective Social Class		Objective Social Class		
Study			*Experimental Manipulation*	*Subjective Rating*	*Education*	*Income*	*Parental Education*
Study 1	face emotion identification (eyes only)	full sample (N = 179)	no significant relationship*	lower social class; better emotion perception	lower social class; better emotion perception**	no significant relationship	n/a
Study 2	face emotion identification (eyes only)	full sample (N_education_ = 2,726; N_income_ = 4,312; N_parent education_ = 4,225)	n/a	n/a	no significant relationship	lower social class; poorer emotion perception	lower social class; poorer emotion perception
		restricted sample^ŧ^ (N_education_ = 990; N_income_ = 1,278; N_parent education_ = 1,267)	n/a	n/a	lower social class; poorer emotion perception	no significant relationship	lower social class; poorer emotion perception
Study 3	face emotion identification (basic emotions	full sample (N = 2,564)	n/a	n/a	lower social class; poorer emotion perception	n/a	n/a
		restricted sample^ŧ^ (N = 1,486)	n/a	n/a	no significant relationship	n/a	n/a
Study 4	face emotion discrimination (basic emotions)	full sample (N_education_ = 2,079; N_income_ = 3,272; N_parent education_ = 3,225)	n/a	n/a	lower social class; poorer emotion perception	no significant relationship	lower social class; poorer emotion perception
		restricted sample^ŧ^ (N_education_ = 953; N_income_ = 892; N_parent education_ = 1,317)	n/a	n/a	lower social class; poorer emotion perception	no significant relationship	lower social class; poorer emotion perception

*Note*: Subjective social class reflects participants’ ladder ratings, regardless of experimental condition.

^ŧ^Restricted sample refers to analyses conducted on individuals self-reporting European/White ethnicity and English as their native language. In Study 2, the sample was further restricted to individuals who reported the United States as their country of origin.

*this null finding should be considered in light of the fact that the experimental manipulation was not successful.

**indicates relationship was only significant when age and age^2^ were included as covariates in addition to gender and vocabulary.

Consistent with prior research, lower subjective social class (i.e., ladder ratings) was associated with higher emotion perception ability, as operationalized by identification of complex facial expressions from the eyes alone (Study 1). Also consistent with prior research, lower scores on one objective social class measure (education) was related to better complex emotion perception (Study 1); however, this relationship was only significant after including certain covariates and was not observed in a larger independent sample (Study 2). Aside from these partial consistencies with prior research, the predicted relations between lower social class and better emotion perception were not observed within multiple larger, more diverse samples when using objective measures of social class (education, income, parental education) and other measures of emotion perception (Studies 3 and 4). Indeed, the opposite pattern of results was observed when using other measures of emotion perception: lower social class was related to poorer performance on emotion identification and discrimination tasks involving basic emotions (Studies 3 and 4). These findings are consistent with findings from other recent studies that examined social class and emotion perception in smaller samples. In one recent study, education and family income did not predict RMET performance [26]. In addition, studies of power, a construct related to social class, suggest that the relationships between higher power and emotion perception are complex and involve interactions with additional variables such as personal beliefs and whether power was obtained legitimately [26–28]. Taken together, our study and others suggest limits to the generalizability of links between social class and emotion perception, and they highlight the need for further work to identify the necessary conditions for such an effect to emerge.

It is important to note that the failure to generalize results of previous research outside of university and laboratory-based settings does not imply the absence of a relationship between social class and emotion perception. Rather, the present findings suggest that the relationship between social status and emotion perception may depend critically upon variable operationalization (e.g., income or education; complex versus basic emotions) and/or the sociodemographic characteristics of the participant sample. As noted, there is little consistency in how social class and emotion perception are operationalized in the literature, and previous studies have typically included only a single measure of each construct. The present study attempted to address these limitations by systematically varying certain variables across studies, including the participant sample, the emotion perception measure, and the measure of social class. However, it is important to note that only one study explored subjective social class and its experimental manipulation was unsuccessful. Therefore, the following conclusions about generalizability relate primarily to objective social class measures. Additional research into subjective social class is necessary.

Taken together, results from the four studies in the present investigation raise the possibility that certain emotion perception tasks may be more sensitive to social class differences than others. We chose the RMET for Studies 1 and 2 because it is a standard, reliable measure of emotion perception [29] involving complex emotions, and may thus be particularly sensitive to subtle group differences. However, vocabulary was a robust predictor of RMET performance in both samples, suggesting that the RMET may measure emotion perception differently among individuals from varying educational backgrounds. In Studies 3 and 4, we explored whether social class was associated with the ability to identify or discriminate between basic emotion expressions. In both studies, lower social class (measured using education variables) was linked to poorer emotion perception. These discrepant findings suggest that the relationship between social status and emotion perception depends critically on how emotion perception is operationalized.

Although the present study used standardized emotion identification and discrimination tasks, prior work on social class and emotion perception has examined participants’ ability to judge a partner’s emotions during interpersonal interactions [1, 5]. Such interactions likely involve more complex and varied emotions than the static images used in the present study. In addition, interpersonal interactions include non-facial behaviors that can serve as clues to the person’s emotional state. As a result, the ability of individuals to judge the emotional states of others during interpersonal interactions may have more ecological validity than the standard tests of emotion identification used in the laboratory and may also be more sensitive to differences in social class. Future research using large and diverse samples can examine whether social class influences emotional perception during interpersonal interactions.

The present findings also suggest that the way social class is operationalized is critical for understanding the relationship between social class and emotion perception. Across studies, the operationalization of social class by participants’ subjective rankings, participants’ education levels, parental education levels, and income resulted in different associations with emotion perception. Future studies comparing and contrasting the predictive abilities of different social class measures on different emotion perception measures are logical and important next steps.

The present findings also raise the possibility that participant demographics influence the relationship between social class and emotion perception. For example, relative to previous studies [1] in which a majority of participants were ethnic minorities and university students, our participants were more diverse in age, country of origin, and educational background. In Studies 2–4, we compared associations between social class and emotion perception in the full sample to test the generalizability of the findings to participants from non-U.S. cultures. Associations between social class and emotion perception were broadly similar between the full sample and the subsample of individuals restricted to represent U.S. and/or White/European individuals. However, it is likely that relations between social class and emotion perception will vary across cultures. This topic warrants further investigation. Perceptions of the self–particularly, of being more or less context-dependent–have been shown to vary across cultural groups [30, 31], and have been proposed to shape perceptual processes [32, 33]. As subjective social class is operationalized as a comparison with one’s social context, it is possible that the effects of subjective social class on emotion perception may be particularly salient among individuals with interdependent views of self. Though a comparison of the associations between social class and emotion perception across specific cultural groups is beyond the scope of the present study, we have made the data for Studies 2–4 available to facilitate future research in this area.

Finally, the present findings indicate that gender, age, agreeableness, and vocabulary should be evaluated in studies of social class and emotion perception. Prior research demonstrates that emotion perception ability changes over time [16], differs between genders [34], and correlates with agreeableness [1]. Conceivably, a relationship with vocabulary could be specific to the RMET; however, existing research on social class and emotion perception has not consistently accounted for each of the other factors. Overall, increased attention to factors that might influence social class and emotion perception would advance the field.

### Limitations

A critical difference between the present and previous studies is the online recruitment and testing of participants. Although aspects of online recruitment–namely, the large and diverse set of participants who differ from the undergraduate participants and university employees used in prior work—represent a strength of the current study, studies indicate that context influences the prosocial behavior of low and high social classes [35]. In particular, the failure to manipulate subjective social class in Study 1 raises the possibility that online priming of subjective social class is less effective than the in-person manipulation of social class used in previous research [1]. Since day-to-day assessments of one’s subjective social class are likely to occur through social interactions, the lab-based priming methods used in previous research [1] may hold more ecological validity than the online manipulation of subjective social class used in the present study. Although existing evidence suggests high comparability of measurement reliability and validity between laboratory-based and online paradigms [20], and though a recent lab-based study also failed to replicate the effect between social class and emotion perception [26], the impact of online testing, especially on experimental manipulation of subjective social class, requires future study.

### Conclusions

The present findings highlight important caveats to the literature linking lower social class with better emotion perception, and suggest that a critical next step for this area of research will be a systematic investigation to discern which measures of social class predict which measures of emotion perception in which populations. Such research would advance the field’s evolving understanding of the mechanisms by which social class may impact emotion perception, and could facilitate investigations of how social class influences other social behaviors (for review see [36]).

## Supporting information

S1 Fig Subjective social class manipulation in Study 1. The subjective social class manipulation was identical to the one used in Kraus et al. (2010) Study 3. Participants viewed a social class ladder and were randomly assigned to compare themselves to someone at the top of the ladder (downward social class comparison; left) or the bottom of the ladder (upward social class comparison; right). Then participants wrote briefly about a hypothetical interaction with that individual and then rated their own subjective social class rating.
(JPG)

S2 Fig Sample stimuli from the reading the mind in the eyes (rmet), famous faces, and vocabulary tasks in Study 1. During the RMET, participants viewed images of the eye region and were asked to identify the emotion being expressed from four choices placed around the image. During the Famous Faces Task, participants viewed images of famous individuals and typed in the name of the individual. During the Vocabulary Task, participants viewed individual words and chose the best definition of the word from four options.
(JPG)

S1 Table The relationship between different measures of social class and rmet performance in Study 1 after accounting for age covariates. (DOCX)

S2 Table Countries represented in the participant samples of studies 2–4. (DOCX)

S3 Table The relationship between different measures of social class and RMET performance in Study 2 after accounting for age covariates. (DOCX)

S4 Table The relationship between different measures of social class and RMET performance in Study 2 After restricting participants to those who reported the United Sates as their Country of Origin, European/White Ethnicity, and English as their Native Language. (DOCX)

S5 Table The relationship between participant education and emotion identification ability in Study 3 After Accounting for gender, age, and age^2^ after restricting participants to those who Reported European/White Ethnicity and English as their Native Language. (DOCX)

S6 Table The relationship between participant education and emotion identification ability in Study 4 after restricting participants to those who reported European/White Ethnicity and English as their Native Language. (DOCX)
