# Supplementary figures and images for: How generalizable is the inverse relationship between social class and emotion perception?
Source: PLoS One. 2018 Oct 19;13(10):e0205949. doi: 10.1371/journal.pone.0205949 (PMC6195285; doi:10.1371/journal.pone.0205949)

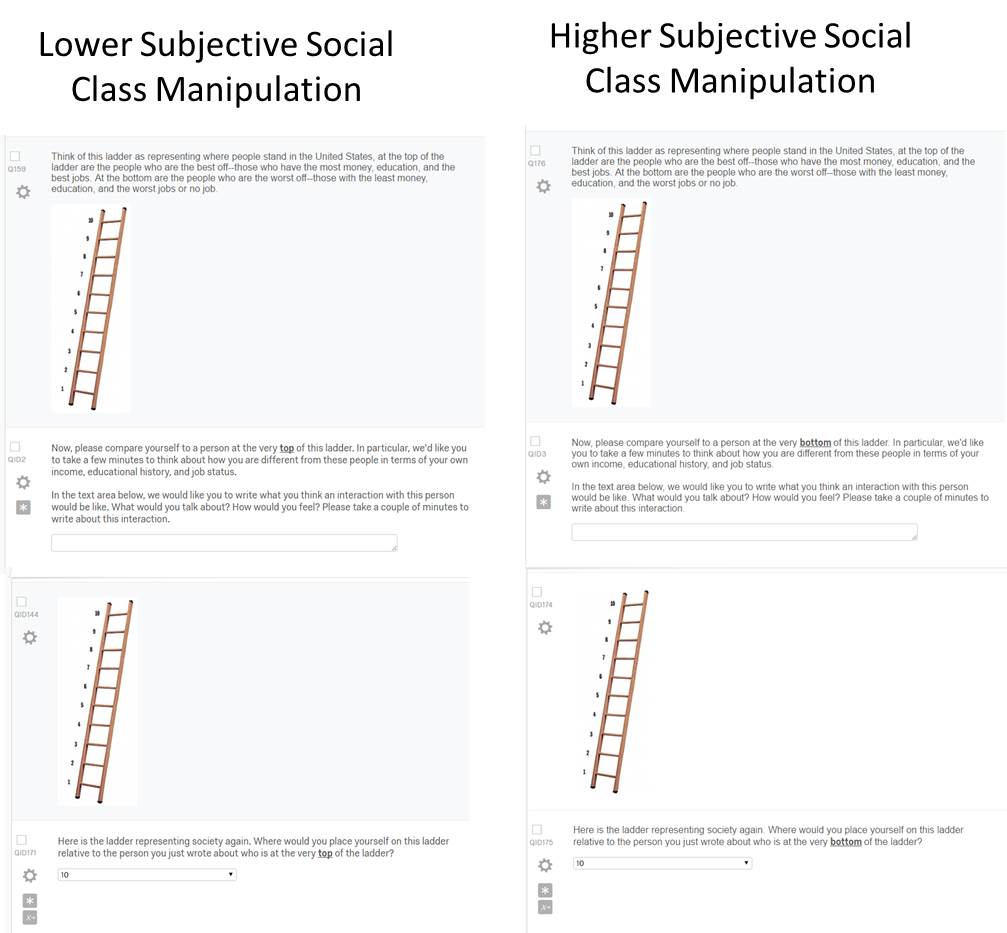

Supplement: S1 Fig — The subjective social class manipulation was identical to the one used in Kraus et al. (2010) Study 3. Participants viewed a social class ladder and were randomly assigned to compare themselves to someone at the top of the ladder (downward social class comparison; left) or the bottom of the ladder (upward social class comparison; right). Then participants wrote briefly about a hypothetical interaction with that individual and then rated their own subjective social class rating. (JPG) [file pone.0205949.s001.jpg]

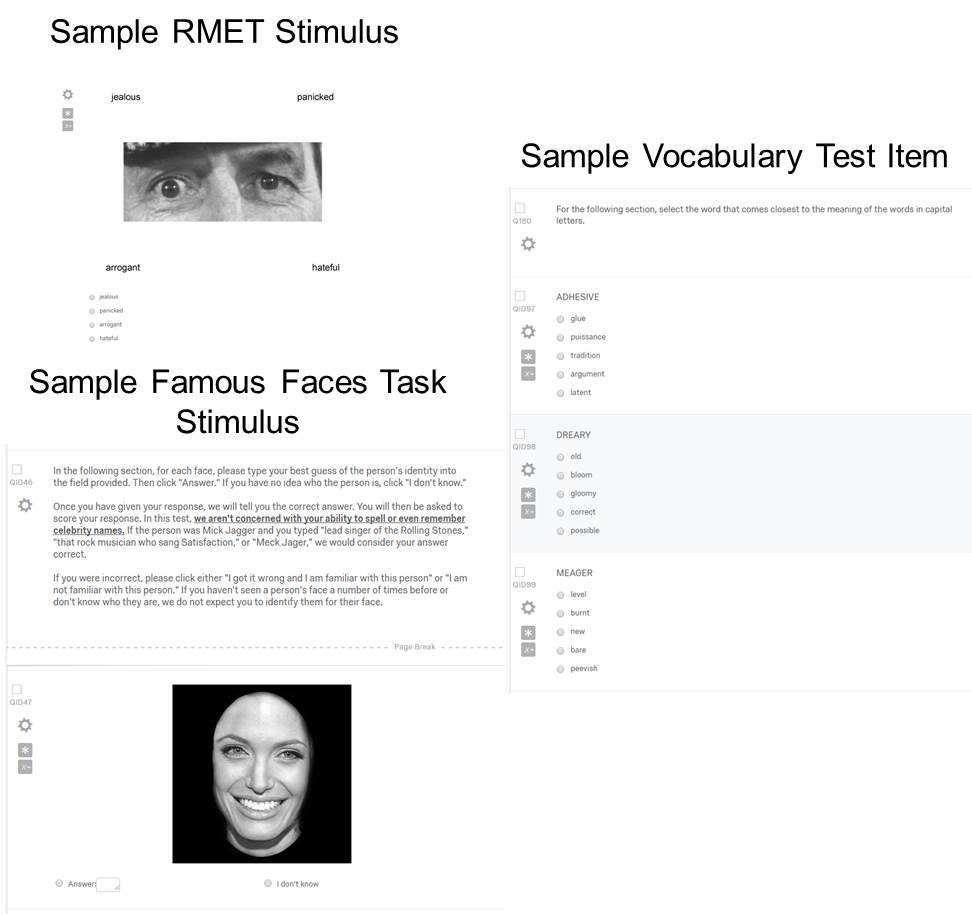

Supplement: S2 Fig — During the RMET, participants viewed images of the eye region and were asked to identify the emotion being expressed from four choices placed around the image. During the Famous Faces Task, participants viewed images of famous individuals and typed in the name of the individual. During the Vocabulary Task, participants viewed individual words and chose the best definition of the word from four options. (JPG) [file pone.0205949.s002.jpg]
