# Supplementary material for: How generalizable is the inverse relationship between social class and emotion perception?
Source: PLoS One. 2018 Oct 19;13(10):e0205949. doi: 10.1371/journal.pone.0205949 (PMC6195285; doi:10.1371/journal.pone.0205949)
Supplement: S1 Table — (DOCX) [file pone.0205949.s003.docx]

S1 Table. *The Relationship Between Different Measures of Social Class and RMET Performance in Study 1 After Accounting for Age Covariates*

| Predictor |  | Subjective Social Class (Ladder Ratings) | Participant Education | Participant Income |
| --- | --- | --- | --- | --- |
| Gender | *B* | 0.53 | 0.77 | 1.28 |
|  | 95% CI | [-0.85, 1.91] | [-0.70, 2.24] | [-0.17, 2.74] |
| Vocabulary | *B* | 0.57*** | 0.64*** | 0.65*** |
|  | 95% CI | [0.38, 0.76] | [0.44, 0.84] | [0.45, 0.85] |
| Agreeableness | *B* | 0.08* | 0.07 | 0.09* |
|  | 95% CI | [0.00, 0.16] | [-0.01, 0.16] | [0.01, 0.17] |
| Age | *B* | 0.54** | 0.55* | 0.65* |
|  | 95% CI | [0.15, 0.92] | [0.04, 1.06] | [0.13, 1.17] |
| Age^2^ | *B* | -0.01* | -0.01 | -0.01* |
|  | 95% CI | [-0.01, 0.00] | [-0.01, 0.00] | [-0.01, 0.00] |
| Social Class | *B* | -0.48* | -1.47* | -0.29 |
|  | 95% CI | [-0.89, -0.07] | [-2.87, -0.07] | [-0.66, 0.08] |
|  | *N* | 179 | 148 | 144 |
|  | R^2^ | 0.31 | 0.29 | .30 |
|  | *F* | 12.69*** | 9.16*** | 9.82*** |

*Note.* CI= confidence interval.

**p* ≤ .05. ** *p* ≤ .01. *** *p* < .001.
