# Supplementary material for: How generalizable is the inverse relationship between social class and emotion perception?
Source: PLoS One. 2018 Oct 19;13(10):e0205949. doi: 10.1371/journal.pone.0205949 (PMC6195285; doi:10.1371/journal.pone.0205949)
Supplement: S2 Table — (DOCX) [file pone.0205949.s004.docx]

Table 4. *Countries Represented in the Participant Samples of Studies 2-4*

|  | Study 2 | Study 3 | Study 4 |
| --- | --- | --- | --- |
| Country^ŧ^ | N (%) | N (%) | N (%) |
| Albania | 11 (0.2) | -- | 6 (0.2) |
| Algeria | 10 (0.2) | -- | 2 (0.1) |
| Argentina | 14 (0.3) | 6 (0.2) | 18 (0.5) |
| Asia* | 19 (0.4) | -- | 8 (0.2) |
| Australia | 126 (2.4) | 165 (6.4) | 153 (4) |
| Austria | 6 (0.1) | 9 (0.4) | 3 (0.1) |
| Bangladesh | 16 (0.3) | 3 (0.1) | 15 (0.4) |
| Belgium | 15 (0.3) | 10 (0.4) | 19 (0.5) |
| Bosnia and Herzegovina | 5 (0.1) | 2 (0.1) | 4 (0.1) |
| Brazil | 37 (0.7) | 18 (0.7) | 43 (1.1) |
| Bulgaria | 19 (0.4) | 6 (0.2) | 31 (0.8) |
| Canada | 215 (4.1) | 149 (5.8) | 153 (4) |
| Chile | 9 (0.2) | 3 (0.1) | 4 (0.1) |
| China | 51 (1) | 12 (0.5) | 112 (2.9) |
| Colombia | 10 (0.2) | -- | 6 (0.2) |
| Croatia | 15 (0.3) | 10 (0.4) | 22 (0.6) |
| Cyprus | 4 (0.1) | 3 (0.1) | 7 (0.2) |
| Czech Republic | 8 (0.2) | 5 (0.2) | 5 (0.1) |
| Denmark | 22 (0.4) | 9 (0.4) | 20 (0.5) |
| Dominician Republic | 8 (0.2) | 2 (0.1) | 2 (0.1) |
| Ecuador | 5 (0.1) | 1 (0) | -- |
| Egypt | 20 (0.4) | 1 (0) | 27 (0.7) |
| England | 323 (6.2) | 303 (11.8) | 195 (5.1) |
| Estonia | 5 (0.1) | 1 (0) | 7 (0.2) |
| Europe* | 5 (0.1) | -- | 6 (0.2) |
| Finland | 26 (0.5) | 6 (0.2) | 29 (0.8) |
| France | 30 (0.6) | 35 (1.4) | 21 (0.5) |
| Germany | 58 (1.1) | 49 (1.9) | 53 (1.4) |
| Greece | 81 (1.6) | 14 (0.5) | 44 (1.1) |
| Holland | 6 (0.1) | 7 (0.3) | 5 (0.1) |
| Hong Kong | 19 (0.4) | 5 (0.2) | 19 (0.5) |
| Hungary | 16 (0.3) | 1 (0) | 29 (0.8) |
| Iceland | 2 (0) | 5 (0.2) | 6 (0.2) |
| India | 600 (11.6) | 87 (3.4) | 293 (7.6) |
| Indonesia | 36 (0.7) | 16 (0.6) | 69 (1.8) |
| Iran | 36 (0.7) | 7 (0.3) | 37 (1) |
| Iraq | 9 (0.2) | 1 (0) | 9 (0.2) |
| Ireland | 35 (0.7) | 15 (0.6) | 25 (0.6) |
| Israel | 10 (0.2) | 13 (0.5) | 19 (0.5) |
| Italy | 70 (1.3) | 16 (0.6) | 26 (0.7) |
| Japan | 8 (0.2) | 6 (0.2) | 9 (0.2) |
| Jordan | 10 (0.2) | 3 (0.1) | 15 (0.4) |
| Kenya | 13 (0.3) | 1 (0) | 3 (0.1) |
| Kuwait | 4 (0.1) | 3 (0.1) | 5 (0.1) |
| Latvia | 8 (0.2) | 3 (0.1) | 8 (0.2) |
| Lebanon | 9 (0.2) | 2 (0.1) | 7 (0.2) |
| Lithuania | 15 (0.3) | 3 (0.1) | 14 (0.4) |
| Macedonia | 16 (0.3) | 2 (0.1) | 5 (0.1) |
| Malaysia | 60 (1.2) | 13 (0.5) | 55 (1.4) |
| Mauritius | 5 (0.1) | 1 (0) | 3 (0.1) |
| Mexico | 33 (0.6) | 12 (0.5) | 77 (2) |
| Morocco | 11 (0.2) | -- | 8 (0.2) |
| Nepal | 7 (0.1) | -- | 4 (0.1) |
| Netherlands | 26 (0.5) | 25 (1) | 20 (0.5) |
| New Zealand | 30 (0.6) | 30 (1.2) | 24 (0.6) |
| Nigeria | 8 (0.2) | 3 (0.1) | 2 (0.1) |
| Norway | 20 (0.4) | 55 (2.1) | 19 (0.5) |
| Pakistan | 90 (1.7) | 16 (0.6) | 63 (1.6) |
| Peru | 5 (0.1) | -- | 8 (0.2) |
| Philippines | 89 (1.7) | 15 (0.6) | 43 (1.1) |
| Poland | 27 (0.5) | 10 (0.4) | 34 (0.9) |
| Portugal | 24 (0.5) | 12 (0.5) | 39 (1) |
| Romania | 84 (1.6) | 18 (0.7) | 50 (1.3) |
| Russia | 14 (0.3) | 11 (0.4) | 10 (0.3) |
| Saudi Arabia | 14 (0.3) | -- | 14 (0.4) |
| Scotland | 27 (0.5) | -- | 21 (0.5) |
| Serbia | 27 (0.5) | 1 (0) | 19 (0.5) |
| Singapore | 17 (0.3) | 15 (0.6) | 18 (0.5) |
| Slovakia | 8 (0.2) | 1 (0) | 7 (0.2) |
| Slovenia | 8 (0.2) | 1 (0) | 8 (0.2) |
| South Africa | 69 (1.3) | 29 (1.1) | 48 (1.2) |
| Spain | 13 (0.3) | 13 (0.5) | 17 (0.4) |
| Sri Lanka | 16 (0.3) | 5 (0.2) | 6 (0.2) |
| Sweden | 44 (0.8) | 41 (1.6) | 28 (0.7) |
| Switzerland | 4 (0.1) | 15 (0.6) | 6 (0.2) |
| Syria | 5 (0.1) | -- | 3 (0.1) |
| Taiwan | 6 (0.1) | 1 (0) | 5 (0.1) |
| Thailand | 11 (0.2) | 10 (0.4) | 5 (0.1) |
| Tunisia | 14 (0.3) | -- | 7 (0.2) |
| Turkey | 32 (0.6) | 19 (0.7) | 35 (0.9) |
| Ukraine | 12 (0.2) | 1 (0) | 4 (0.1) |
| United Arab Emirates | 11 (0.2) | 6 (0.2) | 12 (0.3) |
| United States | 1971 (38) | 1115 (43.4) | 1334 (34.6) |
| Venezuela | 5 (0.1) | 4 (0.2) | 4 (0.1) |
| Vietnam | 15 (0.3) | 3 (0.1) | 23 (0.6) |
| Wales | 12 (0.2) | -- | 8 (0.2) |
| Unable to Classify | 24 (0.5) | -- | 25 (0.6) |
| Multiple Countries | 37 (0.7) | -- | 19 (0.5) |
| No Response | 42 (0.8) | 2 (0.1) | 16 (0.4) |

*Note:* Data for Studies 2 and 4 were based on free text responses to the question “What country did you grow up in?” For Study 3, country of residence was estimated based on the IP addresses of the participants when participating in the study. Unable to Classify = we were unable to identify a specific country based on the participant’s response. Multiple Countries = participant reported growing up in 2 or more countries. The complete data are available at [osf.io/jf7r3](file:///\\ntm.wellesley.edu\Users\lauragermine\Dropbox%20(Personal)\Deveney%20paper\osf.io\jf7r3)

^ŧ^The following countries were reported by fewer than 5 participants in each of the 3 studies: Abu Dhabi, Afghanistan, Africa*, Arab or Middle Eastern*, Armenia, Aruba, Azad Kashmir, Azerbaijan, Bahamas, Bahrain, Belarus, Benin, Bhutan, Bolivia, Brunei Darussalam, Cambodia, Cameroon, Cook Islands, Costa Rica, Cuba, Curacao, El Salvador, Ethiopia, Fiji, Gambia, Georgia, Ghana, Guatemala, Guernsey, Guyana, Haiti, Honduras, Jamaica, Kazakhstan, Korea, Kosovo, Kurdistan, Kyrgyzstan, Liberia, Libya, Luxembourg, Maldives, Malta, Moldova, Monaco, Mongolia, Montenegro, Morocco, Mozambique, Myanmar, Namibia, Norther Mariana Islands, Oman, Palestine, Panama, Paraguay, Qatar, Senegal, Seychelles, Somalia, South America*, South Asia*, South Korea, St. Lucia, Sudan, Suriname, Tajikistan, Tanzania, Trinidad and Tobago, Uganda, Uruguay, Uzbekistan, Yugoslavia, Zambia, Zimbabwe.

*Participants did not provide a specific country within this region.
