# Supplementary material for: How generalizable is the inverse relationship between social class and emotion perception?
Source: PLoS One. 2018 Oct 19;13(10):e0205949. doi: 10.1371/journal.pone.0205949 (PMC6195285; doi:10.1371/journal.pone.0205949)
Supplement: S3 Table — (DOCX) [file pone.0205949.s005.docx]

S3 Table. *The Relationship Between Different Measures of Social Class and RMET Performance in Study 2 After Accounting for Age Covariates*

| Predictor |  | Participant Education | Family Income | Parental Education |
| --- | --- | --- | --- | --- |
| Gender | *B* | 1.39*** | 1.43*** | 1.38*** |
|  | 95% CI | [1.09, 1.69] | [1.18, 1.68] | [1.13, 1.63] |
| Vocabulary | *B* | 0.59*** | 0.58*** | 0.57*** |
|  | 95% CI | [0.55, 0.63] | [0.54, 0.61] | [0.54, 0.61] |
| Age | *B* | 0.04 | 0.04 | 0.06* |
|  | 95% CI | [-0.04, 0.13] | [-0.01, 0.10] | [0.005, 0.11] |
| Age^2^ | *B* | 0.00 | 0.00** | -0.001** |
|  | 95% CI | [-0.002, 0.00] | [-0.002, 0.00] | [-0.02, 0.00] |
| Social Class | *B* | 0.12 | 0.13* | 0.46*** |
|  | 95% CI | [-0.19, 0.42] | [0.01, 0.25] | [0.24, 0.69] |
|  | *N* | 2,726 | 4,312 | 4,225 |
|  | R^2^ | .29 | .27 | .27 |
|  | *F* | 219.61*** | 312.56*** | 318.43*** |

*Note.* CI= confidence interval.

**p* ≤ .05. ** *p* ≤ .01. *** *p* < .001.
