# Supplementary material for: How generalizable is the inverse relationship between social class and emotion perception?
Source: PLoS One. 2018 Oct 19;13(10):e0205949. doi: 10.1371/journal.pone.0205949 (PMC6195285; doi:10.1371/journal.pone.0205949)
Supplement: S4 Table — (DOCX) [file pone.0205949.s006.docx]

S4 Table. *The Relationship Between Different Measures of Social Class and RMET Performance in Study 2 After Restricting Participants to those who Reported the United Sates as their Country of Origin, European/White Ethnicity, and English as their Native Language*

| Predictor |  | Participant Education | Family Income | Parental Education |
| --- | --- | --- | --- | --- |
| Gender | *B* | 0.98*** | 1.09*** | 1.13*** |
|  | 95% CI | [0.52, 1.44] | [0.69, 1.50] | [0.73, 1.53] |
| Vocabulary | *B* | 0.40*** | 0.44*** | 0.42*** |
|  | 95% CI | [0.32, 0.48] | [0.37, 0.51] | [0.35, 0.49] |
| Age | *B* | 0.00 | -0.05 | -0.04 |
|  | 95% CI | [-0.11, 0.11] | [-0.12, 0.03] | [-0.11, 004] |
| Age^2^ | *B* | 0.00 | 0.00 | 0.00 |
|  | 95% CI | [-0.001, 0.001] | [-0.001, 0.001] | [-0.001, 0.001] |
| Social Class | *B* | 0.51* | 0.13 | 0.59** |
|  | 95% CI | [0.05, 0.97] | [-0.06, 0.31] | [0.22, 0.96] |
|  | *N* | 990 | 1,278 | 1,267 |
|  | R^2^ | .13 | .14 | .15 |
|  | *F* | 30.46*** | 41.97*** | 44.63*** |

*Note.* CI= confidence interval.

**p* ≤ .05. ** *p* ≤ .01. *** *p* < .001.
