# Supplementary material for: How generalizable is the inverse relationship between social class and emotion perception?
Source: PLoS One. 2018 Oct 19;13(10):e0205949. doi: 10.1371/journal.pone.0205949 (PMC6195285; doi:10.1371/journal.pone.0205949)
Supplement: S5 Table — (DOCX) [file pone.0205949.s007.docx]

S5 Table. *The Relationship Between Participant Education and Emotion Identification Ability in Study 3 After Restricting Participants to those who Reported European/White Ethnicity and English as their Native Language*

| Predictor |  | Participant Education |
| --- | --- | --- |
| Gender | *B* | 0.70** |
|  | 95% CI | [0.30, 1.10] |
| Age | *B* | 0.05 |
|  | 95% CI | [-.05, 0.15] |
| Age^2^ | *B* | -0.001 |
|  | 95% CI | [-0.002, 0.00] |
| Social Class | *B* | 0.38 |
|  | 95% CI | [-0.01, 0.77] |
|  | *N* | 1,486 |
|  | R^2^ | .02 |
|  | *F* | 6.44*** |

*Note.* CI= confidence interval.

**p* ≤ .05. ** *p* ≤ .01. *** *p* < .001.
