# Supplementary material for: How generalizable is the inverse relationship between social class and emotion perception?
Source: PLoS One. 2018 Oct 19;13(10):e0205949. doi: 10.1371/journal.pone.0205949 (PMC6195285; doi:10.1371/journal.pone.0205949)
Supplement: S6 Table — (DOCX) [file pone.0205949.s008.docx]

S6 Table. *The Relationship Between Different Measures of Social Class and Emotion Discrimination Ability in Study 4 After Restricting Participants to those who Reported European/White Ethnicity and English as their Native Language*

| Predictor |  | Participant Education | Family Income | Parental Education |
| --- | --- | --- | --- | --- |
| Gender | *B* | 1.05** | 1.17** | 0.85** |
|  | 95% CI | [0.39, 1.71] | [0.48, 1.86] | [0.29, 1.41] |
| Age | *B* | 0.16 | 0.16 | 0.09 |
|  | 95% CI | [0.00, 0.32] | [-.01, 0.32] | [-.01, 0.19] |
| Age^2^ | *B* | -0.002 | -0.001 | -0.001 |
|  | 95% CI | [-0.003, 0.00] | [-0.003, 0.00] | [-0.002, 0.001] |
| Identity Discrimination | *B* | 0.30*** | 0.29*** | 0.31*** |
|  | 95% CI | [0.25, 0.35] | [0.24, 0.35] | [0.27, 0.36] |
| Social Class | *B* | 1.20*** | 0.06 | 0.68** |
|  | 95% CI | [0.59, 1.81] | [-0.27, 0.38] | [0.19, 1.18] |
|  | *N* | 953 | 892 | 1,317 |
|  | R^2^ | .16 | .14 | .15 |
|  | *F* | 36.27*** | 28.67*** | 47.57*** |

*Note.* CI= confidence interval.

**p* ≤ .05. ** *p* ≤ .01. *** *p* < .001.
